# Latent EBV reactivation drives aberrant B-cell proliferation during ex vivo tumor-infiltrating lymphocyte expansion from EBV-negative rectal cancer tumor tissue

**DOI:** 10.1038/s41598-025-29456-7

**Published:** 2025-11-24

**Authors:** Tatiana V. Petrova, Daria V. Kuznetzova, Alexandra V. Kanygina, Liubov O. Skorodumova, Viktor A. Ivanov, Tatiana A. Astrelina, Svetlana E. Varlamova, Elena A. Zerkalenkova, Elena I. Sharova

**Affiliations:** 1 Lopukhin FRCC PCM, 1a Malaya Pirogovskaya St, Moscow, 119435 Russia; 2 The Burnasyan Federal Medical Biophysical Center FMBA of Russia, Moscow, Russia; 3 Dmitry Rogachev National Scientific and Practical Center of Pediatric Hematology, Oncology and Immunology, Moscow, Russia

**Keywords:** Immunotherapy, Rectal cancer, Herpes virus

## Abstract

Recent studies underscore herpesvirus-associated risks, especially HHV6 and Epstein–Barr virus (EBV), in CAR-T and tumor-infiltrating lymphocyte (TIL) therapies, partly due to ex vivo cell manipulation. EBV-driven B cell outgrowth can occur during TIL expansion, and EBV-transformed B cells may exert immunomodulatory functions, potentially impacting TIL therapy efficacy and safety — areas that remain underexplored. Here, we characterized a spontaneously arising wild-type EBV-transformed B cell line, lcl_burn0214, established during ex vivo culture of a tumor specimen from a rectal cancer patient who was EBV-negative. The lcl_burn0214 cell line has undergone 70 passages and was confirmed as a monoclonal B cell line of patient origin via HLA genotyping, immunophenotyping (CD19, CD20), B cell receptor clonotype analysis. RNA-seq analysis revealed that lcl_burn0214’s gene expression closely resembles that of lymphoblastoid cells (*r* = 0.88) rather than malignant B lymphomas. In immunocompromised mice, lcl_burn0214 exhibited limited tumorigenicity. Autologous TILs were negative for B cells and exhibited a strong interferon-gamma response in the ELISPOT assay during coculture with lcl_burn0214 or with the EBV peptide pool (1000–1800 spots per million of TILs). Our findings underscore the importance of monitoring TIL products for EBV-positive B cell contamination during manufacturing to ensure safety and therapeutic efficacy even for EBV-negative patients.

**Supplementary Information:**

The online version contains supplementary material available at 10.1038/s41598-025-29456-7.

## Introduction

EBV, a member of the herpesvirus family, primarily infects B lymphocytes and persists as an episome throughout the host’s lifespan, evading T cell-mediated immune responses. EBV infection is associated with B cell lymphomas and can trigger lymphoproliferative disease in immunocompromised individuals or in those who are immunosuppressed following transplantation of solid organs or hematopoietic cells^1^.

Recent studies have raised concerns about herpesvirus-associated risks in T cell therapies, including chimeric antigen receptor (CAR) T cell and tumor-infiltrating T lymphocyte (TIL) treatments. Both therapies require pre-treatment lymphodepletion to reduce the patients’ existing T cells, including herpesvirus-specific T cells. Latent human herpesvirus 6 reactivation has been demonstrated in CAR-T cells in vivo, along with a reported case of EBV-positive B lymphoma following TIL therapy for metastatic melanoma^2,3^. Several studies have reported the spontaneous ex vivo outgrowth of immortalized EBV-transformed B cell lines derived from various solid and hematological tumors, including ovarian cancer, colorectal cancer, and leukemia^4–6^. Additionally, several animal studies have demonstrated that highly immunocompromised mice engrafted with patient tumor tissues — including ovarian, gastric, breast, lung, and colorectal tumors — often develop metastatic EBV-positive B cell lymphocytic tumors rather than patient-derived epithelial tumor xenografts^7–10^. Tumor fragments that are used to generate cell products for autologous TIL therapy contain diverse cell populations, such as TILs and tumor-infiltrating B lymphocytes (B-TILs)^11^. Therefore, considering above-mentioned studies ex vivo TIL expansion for cell therapy may be associated with the potential risk of concurrent proliferation of EBV-transformed B-TILs, particularly in colorectal cancer, where EBV-positive lymphocyte infiltration of the tumor occurs in approximately 40% of cases^12^. Moreover, EBV-transformed B cells have the potential to modulate immune responses by reshaping T cell receptor repertoire^10^. Finally, as TIL therapy includes a lymphodepletion before the infusion of the TIL cell product, EBV-driven B-TIL uncontrolled proliferation can occur, resulting in a malignant lymphoproliferative disorder. These characteristics of EBV-transformed B-TILs may significantly impact the efficacy and safety of TIL therapies but remain insufficiently explored. Therefore, studying the features of wild-type EBV-transformed B-TIL cell lines derived from tumor tissues during ex vivo culture, as well as their interactions with autologous TILs, is of interest due to the expanding clinical use of TIL therapy. Nevertheless, only one study to date has explored this issue in a breast cancer patient^10^. Here, we characterized a wild-type EBV-transformed immortalized B cell line, (named lcl_burn0214), derived spontaneously from the tumor tissue sample of a patient with rectal adenocarcinoma during ex vivo culture and assessed its tumorigenic potential in immunocompromised mice. Using RNA-seq, we compared its gene expression with established EBV-positive and EBV-negative B lymphoma lines and B95-8 EBV-infected lymphoblastoid cell lines (named B95-8_LCLs) to better position it within the lymphoblastoid-lymphoma cell continuum. We also examined the presence of EBV DNA in tumor, PBMC, TIL, and lcl_burn0214 patient’s samples to determine whether it is possible to predict ex vivo EBV-mediated lymphoproliferation based on the analysis of a patient’s PBMC. Finally we assessed the ability of autologous tumor infiltrating T lymphocytes (named TIL_burn0214) to counteract lcl_burn0214 ex vivo by coculturing the autologous TIL with either lcl_burn0214 or an EBV HLA class I peptide pool, followed by analysis of IFN-gamma production using an ELISPOT assay. Additionally, we evaluated the presence of B cell contamination and EBV DNA in the autologous TIL_burn0214 cell product.

## Results

### Establishment of lcl_burn0214 cell line

The tumor tissue sample, named tum_burn0214, was obtained during the surgery of a 46-year-old Caucasian male diagnosed with microsatellite-stable, moderately differentiated (T4N0M0 G2) rectal adenocarcinoma at the Burnasyan Federal Medical Biophysical Center FMBA of Russia.

**Fig. 1:**
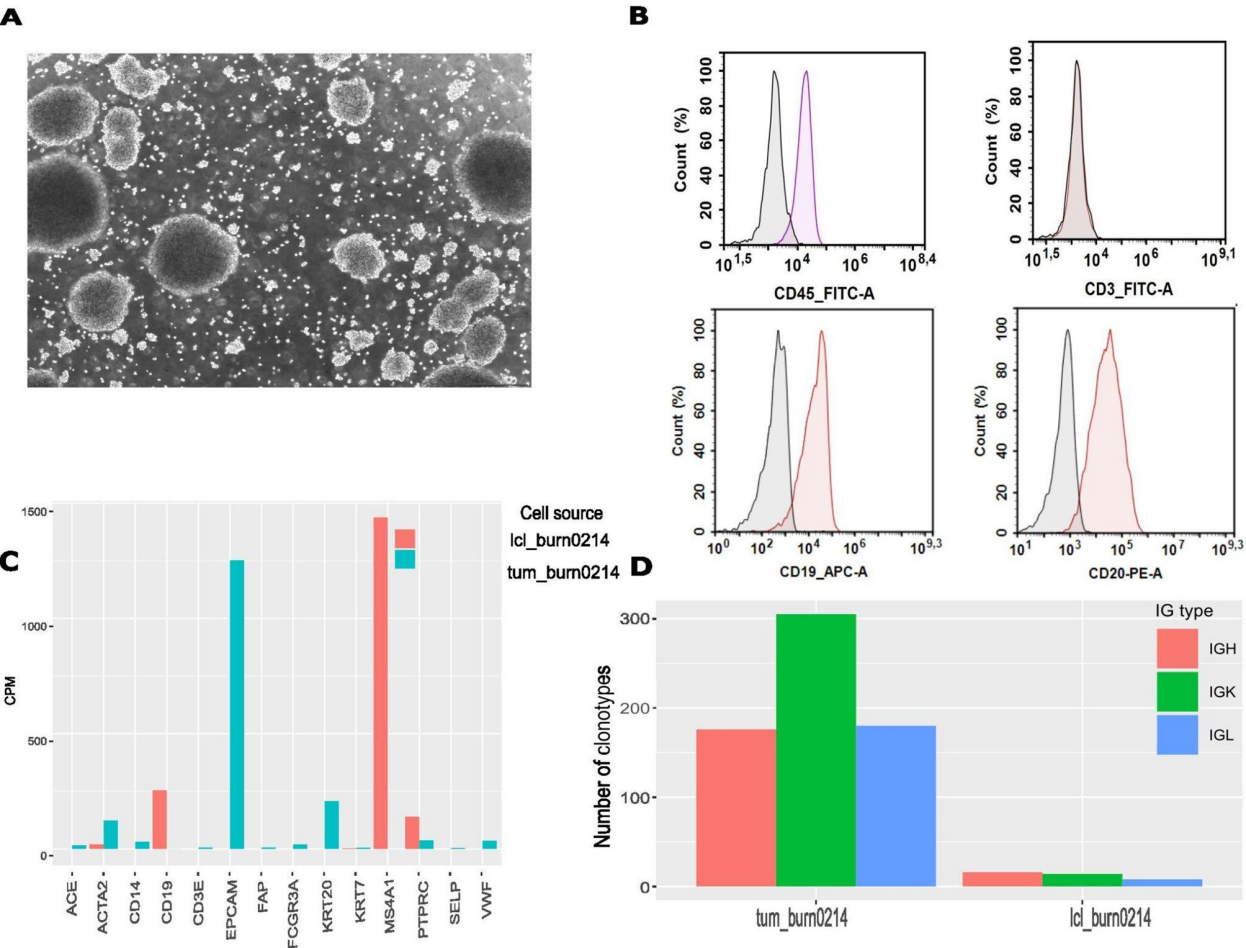
Characteristics of lcl_burn0214 cell line and the tum_burn0214 specimen. **A** Representative bright field image of lcl_burn0214 cell line morphology (scale bar 100 μm). **B** Flow cytometry. Surface expression of CD45, CD19, CD20, CD3 markers in lcl_burn0214 cell line. Cells were counterstained with DAPI to exclude dead cells from analysis. Isotype controls are colored black on histograms, CD45, CD3, CD19, CD20 markers are colored red on histograms. **C** Selected epithelial, endothelial, fibroblast and hematopoietic expression markers in lcl_burn0214 and tum_burn0214 samples. Bars represent gene expression level in CPM (count per million). **D** Number of clonotypes for BCR chains identified in tum_burn0214 and lcl_burn0214 samples. Bars represent the number of IGH, IGK and IGL clones.

Fresh tumor tissue was minced and digested with collagenase IV, and the resulting cell suspension was cultured. Over the following weeks, lymphocyte-like cell clusters formed in suspension. The resulting cell line, lcl_burn0214, has undergone 70 passages (approximately 180 divisions), consistently forming floating clusters. (Fig. 1A). The doubling time of the lcl_burn0214 cell line at passage 49 was determined to be 36 h.

### Characterization of lcl_burn0214 cell line

We first characterized the cell type, heterogeneity, authenticity, and patient origin of lcl_burn0214 cell line. Immunophenotyping revealed CD45+, CD19+, and CD20 + expression, with no CD3 detected (Fig. 1B). At the same time, epithelial, endothelial, and fibroblast markers were minimally expressed in the lcl_burn0214 relative to the tum_burn0214 sample, confirming its B cell identity (Fig. 1C). We observed a significant reduction in B cell receptor clonotype diversity in the emerging lcl_burn0214 cell line relative to the parental tumor, with the proportion of the most frequent BCR clonotype reaching 92–99%, compared to 10–28% in the original tumor tissue specimen tum_burn0214, indicating the near monoclonal nature of the lcl_burn0214 cell line (Fig. 1D, Supplementary Table 1). Due to low B cell infiltration in the tum_burn0214 (Supplementary Table 2 A–C), we were unable to detect any shared clonotypes between the two samples.

HLA genotyping of tum_burn0214 and lcl_burn0214 samples, along with short tandem repeat profiling (STR) of lcl_burn0214, peripheral blood mononuclear cells (PBMC_burn0214), and TIL_burn0214 samples, confirmed their genetic relationship and excluded the potential occurrence of the lcl_burn0214 cell line as a result of cross-contamination with established laboratory cell lines (Supplementary Tables 3, 4). Furthermore, no related cell lines were found among 8,808 human cell lines in the Cellosaurus database (Release 52 of April 2025). Karyotyping of the cell line performed at passage 62 revealed a hyperploid male karyotype with trisomies of chromosomes 9, 12, and 15 in 100% of cells of the lcl_burn0214 cell line. In contrast, at early passage 6, cells exhibited a normal 46,XY karyotype (Supplementary Fig. 1A, B). Thus, lcl_burn0214 appeared to be an authentic, nearly monoclonal B-cell line of patient origin, exhibiting spontaneous and active proliferation that was not caused by karyotypic abnormalities.

### EBV status and tumorigenic potential of the lcl_burn0214 cell line

Immortalised B cell lines can arise from diverse origins, notably, the uncontrolled ex vivo proliferation of B lymphocytes is frequently linked to EBV infection, neoplastic transformation, or a combination of both^13,14^. Consequently, we investigated the EBV status and tumorigenic potential of the established lcl_burn0214 cell line. RNA-seq data detected EBV gene expression in the lcl_burn0214 sample but not in the tum_burn0214 sample (110,100 reads versus zero reads mapped to the EBV genome, respectively). Negative EBV results for the patient’s PBMC, TIL and tumor DNA, and a positive result for the lcl_burn0214 cell line DNA, were confirmed by validated real-time PCR testing performed at a clinical laboratory facility (Supplementary Table 5).

**Fig. 2:**
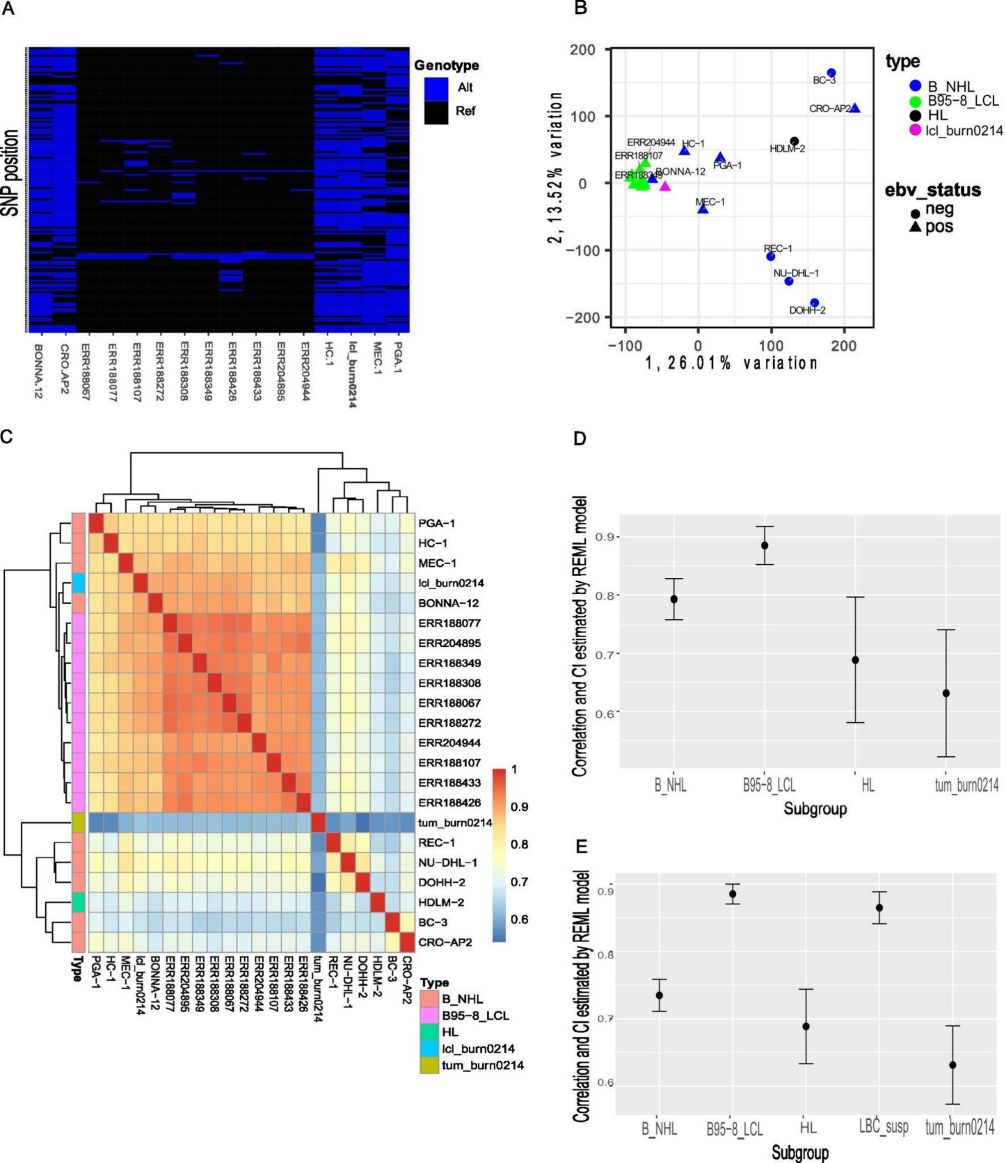
Bulk RNA-seq data analysis of lcl_burn0214, 10 B95-8_LCLs, 5 EBV + and 5 EBV- B cell lymphoma cell lines. **A** Hierarchical clustering of the SNP sites across the EBV genome extracted from Rna-seq data of 10 lymphoblastoid cells obtained by infection PBMC with B95-8 EBV strain (B95-8_LCLs) from Geuvadis consortium dataset, 5 EBV + lymphoma cell lines infected with wild-type EBV from Quentmeier H. dataset and spontaneous lcl_burn0214 cell line. Each row in the heatmap represents an SNP position, and each column represents a cell line. Reference alleles are colored dark blue, while non-reference alleles are colored light blue. The reference genome sequence is the type 1 EBV surrogate genome sequence (NC_007605), constructed based on the B95-8 and Raji EBV genomes. **B** Similarity of malignant and non-malignant B cell lines by principal component analysis of the following samples: B95-8_LCLs, lcl_burn0214, EBV-negative and EBV-positive lymphomas. **C** Similarity of malignant and non-malignant B cell lines by hierarchical clustering of transcriptome correlation of the following samples: B95-8_LCLs, tum_burn0214, lcl_burn0214, EBV-negative and EBV-positive lymphomas. The correlation plot is symmetric on one diagonal. Correlation is pointed by color the more red the greater correlation between the two samples. Cell lines are color-coded according to their cellular origin: B_NHL, green; HL, orange; B95-8_LCLs, blue; lcl_burn0214, violet. **D**,** E** Box plots show the estimated correlation between groups of samples with lcl_burn0214. Pearson correlation coefficients and 95% confidence intervals were estimated using a random effects mixed likelihood (REML) model. The best model for measuring the correlation was the full model (E), which includes the following groups or samples: the B95-8_LCL group; the LBC_susp group, consisting of the Bonna-12, HC-1, MEC-1, and PGA-1 cell lines; the B_NHL group, consisting of the REC-1, NU-DHL-1, DOHH-2, BC-3, and CRO-AP2 cell lines; the HL cell line HDLM-2; and the tum_burn0214 sample. Lcl_burn0214 showed the strongest correlation with the B95-8_LCL group (*r* = 0.88 [0.87–0.90]) and the LBC_susp group (*r* = 0.86 [0.84–0.89]). Correlations with the B_NHL, HL, and tum_burn0214 samples were 0.73 [0.71–0.76], 0.69 [0.63–0.74], and 0.63 [0.57–0.69], respectively. The reduced model (D) includes the following groups or samples: the B95-8_LCL group; the B_NHL group, consisting of the REC-1, NU-DHL-1, DOHH-2, BC-3, and CRO-AP2 cell lines, along with the Bonna-12, HC-1, MEC-1, and PGA-1 cell lines; the HL cell line HDLM-2; and the tum_burn0214 sample.

To exclude the possibility that lcl_burn0214 was infected with the laboratory EBV B95-8 strain, we looked at single nucleotide polymorphisms (SNPs) across the EBV reference genome. RNA-seq analysis of 10 B95-8_LCLs (Geuvadis consortium dataset) and 5 wild-type EBV infected bona fide B lymphoma cell lines (HC-1, MEC-1, PGA-1, CRO-AP2, Bonna-12 from Quentmeier H. dataset) revealed SNP patterns in lcl_burn0214 and B lymphoma cell lines, differing from B95-8_LCLs, which, in turn, closely matched the reference EBV genome (Fig. 2A). This indicates infection of the lcl_burn0214 cell line with wild-type EBV.

We then evaluated the tumorigenic potential of the lcl_burn0214 cell line in NU–A/A Tyrc/Tyrc Foxn1nu/Foxn1nu mice. Initial growth was observed in four out of five mice. In three mice, grafts regressed over a period of 34 days. In one mouse, the graft continued to grow until day 62, reaching a tumor volume of 200 mm³, after which it started regressing (Supplementary Fig. 2A, B). The tumor was then excised. Histological analysis of the excised tumor revealed extensive necrosis with few viable blasts pointing at low tumorigenic potential of the cell line (supplementary Fig. 2C).

### Gene expression analysis of the lcl_burn0214 cell line alongside lymphoblastoid and non-Hodgkin lymphoma cell lines

To better position the lcl_burn0214 cell line within the lymphoblastoid–lymphoma spectrum we analyzed gene expression in the lcl_burn0214 cell line alongside EBV B95-8 infected lymphoblastoid B cell lines from the Geuvadis dataset and bona fide B lymphoma cell lines from the Quentmeier dataset.

We selected RNA-seq data of 10 B95-8_LCLs, and 4 EBV-positive and 5 EBV-negative bona fide B lymphomas, including 1 Hodgkin cell line (HL) (HDLM-2) and 8 non-Hodgkin cell lines (B-NHL) (REC-1, NU-DHL-1, DOHH-2, HDLM-2, BC-3, Bonna-12, HC-1, MEC-1, PGA-1) from white males to minimize sex and race discrepancies (Supplementary Table 6). Principal component analysis and hierarchical clustering (Fig. 2B, C) revealed two major clusters. Notably, the lcl_burn0214, along with the following B-NHL subsets—hairy cell leukemia (HCL) cell lines Bonna-12 and HC-1, and prolymphocytic leukemia (PLL) cell lines MEC-1 and PGA-1—clustered with B95-8_LCLs. In contrast, five other EBV-negative B lymphoma cell lines (REC-1, NU-DHL-1, DOHH-2, HDLM-2, BC-3) and one EBV-positive B lymphoma cell line (CRO-AP2) formed a separate cluster, indicating that clustering was independent of EBV infection, as exemplified by the primary effusion lymphoma (PEL) cell lines BC-3 and CRO-AP2 with differing EBV statuses. Additionally, we measured the correlation of lcl_burn0214 with the groups of B95-8_LCLs, HL cell line, and Non-Hodgkin lymphoma B_NHL cell lines (Fig. 2D, E) using the restricted maximum likelihood (REML) model (Supplementary Table 7). It can be seen that lcl_burn0214 correlated best with the B95-8_LCL group (*r* = 0.88 [0.87–0.90]) and with the Bonna-12, HC-1, MEC-1 and PGA-1 cell lines when these were separated from the B-NHL cell line group into a distinct group named LBC_susp (*r* = 0.86 [0.84–0.89]) (Fig. 2D, E). In contrast, correlations with the group of B_NHL cell lines (REC-1, NU-DHL-1, DOHH-2, HDLM-2, BC-3, CRO-AP2), HL cell line, and tum_burn0214 were lower, with values of 0.73 [0.71–0.76], 0.69 [0.63–0.74], and 0.63 [0.57–0.69], respectively (Fig. 2E).

Collectively, our data show that the established lcl_burn0214 B cell line is lymphoblastoid in nature, is infected with a wild-type EBV strain, and exhibits limited tumorigenicity.

### B cell contamination and specific anti-EBV activity of autologous tumor-infiltrating T lymphocytes TIL_burn0214

**Fig. 3:**
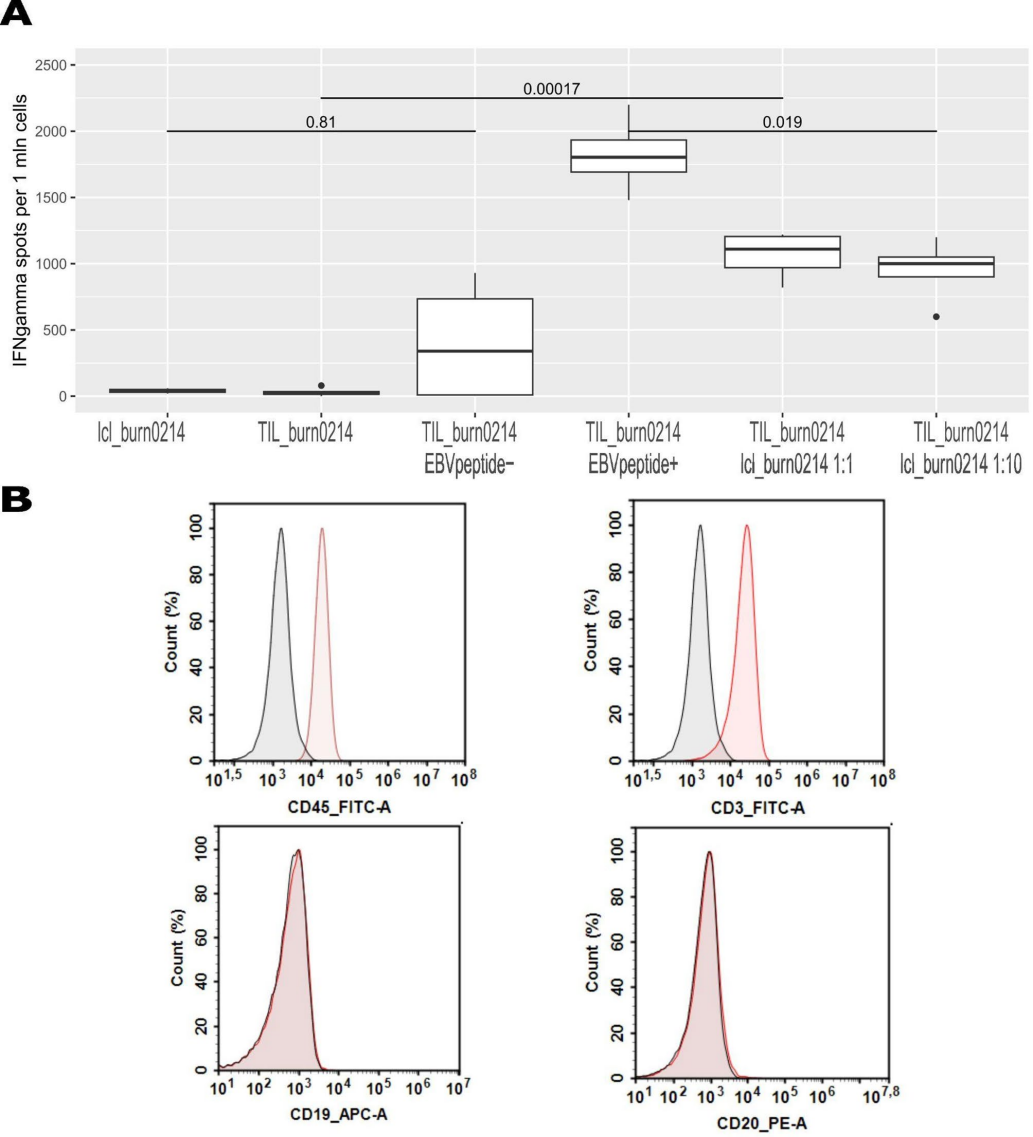
Characteristics of TIL_burn0214. **A** Frequency of TIL_burn0214 cells producing interferon γ (IFN-γ) in response to coculture with lcl_burn0214 or in response to the EBV HLA Class I Peptide Pool. Two vials of TIL_burn0214 were used in co-culture assay as independent biological replicates and run in duplicate wells as technical repeats. TIL_burn0214 cells and the lcl_burn0214 cell line were co-cultured at effector-to-target (E: T) ratios of 1:1 and 1:10. Box plots represent the distribution of IFN-γ spots (representing IFN-γ-producing T cell clones), the horizontal line inside the box shows the median number of IFN-γ spots per 1 mln of TIL_burn0214 or lcl_burn0214 (when TIL_0214 are not added to cell culture). Kruskal-Wallis rank test was used to assess statistically significant differences among the testing groups in the ELISPOT assay. A statistically significant difference (p-value less than 0.05 (= 0.00017)) was observed between the group where lcl_burn0214 or the EBV peptide pool was not added to TIL_burn0214 in comparison to the group where either lcl_burn0214 or the EBV peptide pool was added. Statistically significant difference (p-value less than 0.05) was found within the group where either the EBV peptide pool or lcl_burn0214 was added to TIL_burn0214 (*p* = 0.019). No statistically significant difference (p-value greater than 0.05) was found within the group where TIL_burn0214 were incubated without peptides or lcl_burn0214, and where only lcl_burn0214 was present in the assay (*p* = 0.81). **B** Flow cytometry. Surface expression of CD45, CD19, CD20, CD3 markers in TIL_burn0214. Cells were counterstained with DAPI to exclude dead cells from analysis. Isotype controls are colored black on histograms, CD45, CD3, CD19, CD20 markers are colored red on histograms.

To assess the presence of EBV-specific clones among TIL_burn0214 as a marker of the TILs’ ability to counteract EBV-infected autologous B cells, we conducted a cocultivation assay with lcl_burn0214, as well as an incubation of TIL_burn0214 with an EBV HLA Class I peptide pool. Reactivity to EBV-infected lcl_burn0214 and the EBV peptide pool was evaluated using IFN-γ ELISPOT assay (Fig. 3A). Interestingly, the number of spots per million of TIL_burn0214 — and therefore the corresponding EBV-specific T cell clones detected by the IFN-γ ELISPOT assay — was significantly higher when TIL_burn0214 cells were incubated with EBV HLA class I peptides compared to cocultivation with the autologous lcl_burn0214 cell line. However, in both cases, the frequency of EBV-specific clones did not exceed 0.18% of total TIL_burn0214. With knowledge of the patient’s HLA genotype and the list of EBV peptides in the EBV HLA Class I peptide pool, we narrowed the list of targets to the four EBV peptides potentially specific to TIL_burn0214 derived from the BRLF1, EBNA-3A, EBNA-3C proteins (Supplementary Tables 4, 8).

Finally, we evaluated whether TIL_burn0214 expanded from the rectal tumor specimen tum_burn0214 were contaminated with B cells, using immunophenotyping. The TILs were positive for CD45, CD3 markers but negative for the CD19, CD20 markers (Fig. 3B). As mentioned earlier, PCR analysis confirmed that TIL_burn0214 were EBV-negative (supplementary Table 5). The results indicate no B cell contamination in the TIL_burn0214 culture and the presence of EBV-specific T cell clones.

## Discussion

EBV-driven B cell outgrowth, caused by the virus residing in B cells since early-life infection, poses a potential risk during TIL expansion from tumor tissues ex vivo. Moreover, EBV-transformed B cells may modulate immune responses by altering T cell receptor repertoires, promoting regulatory T cell proliferation, and eventually cause malignant lymphoproliferative disorders in vivo, including in patients who have undergone CAR-T or TIL therapy^1,4,10,15–18^. These factors can ultimately affect the efficacy and safety of TIL therapy—areas that remain largely underexplored. Therefore studying the characteristics of wild-type EBV-transformed B cell lines spontaneously arising from tumor tissues during ex vivo culture, and their interactions with autologous TILs is of interest in light of the growing clinical application of TIL therapy. To our knowledge, only one study, on a breast cancer patient, has examined the relationship between TILs and an autologous wild-type EBV immortalized B cell line, focusing on how the EBV-transformed B cells shape the T cell receptor repertoire of autologous TILs^10^.

Here, we describe the spontaneous emergence of a wild-type EBV-transformed B cell line, lcl_burn0214, from tumor tissue specimen of a patient with microsatellite-stable, moderately differentiated (T4N0M0 G2) rectal adenocarcinoma during ex vivo culture. We characterize the emerging cell line and explore the ability of autologous TILs to counteract potential EBV-driven B cell proliferation during ex vivo culture by assessing the presence of EBV-specific T cell clones as a surrogate marker. The emerged cell line formed lymphocyte-like clusters, grew in suspension, and underwent approximately 180 divisions. Immunophenotyping confirmed its B cell identity (CD45+, CD19+, CD20+, CD3–), with no expression of epithelial, endothelial, or fibroblast marker genes detected. The B cell receptor clonotype diversity in the lcl_burn0214 cell line was an order of magnitude lower compared to the tum_burn0214 sample, pointing at the almost monoclonal nature of the emerging cell line. Schina, A. and colleagues showed that BCR richness in colon adenocarcinoma is relatively low compared to other epithelial tumors, with a maximum of 150–300 BCR clonotypes per tumor specimen^19^. This is consistent with the overall number of BCR clonotypes we observed in the original tumor tissue specimen (tum_burn0214). Furthermore, we did not find shared BCR clonotypes between the tumor sample tum_burn0214 and the lcl_burn0214 cell line. This is likely due to the low initial infiltration of lymphoid cells and the rarity of EBV-transformed B-TILs with the BCR clonotype of interest themselves in the original tumor specimen. It is worth noting that similar spontaneous outgrowth of B cells during ex vivo tumor culture has been reported previously for ovarian, gastric, rectal tumors, and leukaemia^4–6^.

The observed karyotype evolution and chromosomal instability during long-term culturing of the lcl_burn0214 cell line are in concordance with reports from other authors who studied EBV-transformed lymphoblastoid B cell lines^20^. Notably, the frequently encountered trisomy of chromosome 12 in lymphoblastoid cell lines was also observed in our case. Our findings indicate that spontaneous and active proliferation was not initially caused by karyotypic abnormalities, and that the lcl_burn0214 cell line exhibited growth characteristics and clonal evolution typical of EBV-infected B lymphoblastoid cell lines.

Further SNP pattern analysis of the RNA-seq data identified infection of the lcl_burn0214 with a wild-type EBV strain. Unlike the lcl_burn0214 cell line, both the tumor specimen tum_burn0214 and the patient’s PBMCs tested negative for EBV DNA. These observations are in line with previous studies reporting that EBV is not consistently detected in PBMCs of EBV-positive carriers or in tumor samples from EBV-positive nasopharyngeal carcinoma patients. They underscore the rarity of natural EBV-infected B cells in the original tumor tissue and the challenges of predicting EBV-mediated lymphoproliferation both ex vivo and in vivo^21,22^.

Immortalized B cell lines derived from tumor tissue specimens of patients with various cancers have been inconsistently designated by different authors as lymphomas, lymphocytic tumors, or B lymphoblastoid cell lines, reflecting variability in their classification and origin^7–10^. To objectively evaluate the nature of our cell line, we performed a comparative gene expression analysis of the lcl_burn0214 line against lymphoblastoid cell lines and bona fide non-Hodgkin lymphoma cell lines from two external RNA-seq datasets. The hierarchical clustering and correlation analysis supported the lymphoblastoid nature of lcl_burn0214, as it clustered with B95-8_LCLs. But also lcl_burn0214 along with B95-8_LCLs clustered with certain B-NHL cell lines (Bonna-12, HC-1, MEC-1, PGA-1) rather than with B cell lymphomas. The observed clustering patterns align with previous findings that question the authenticity and tumor origin of the cell lines Bonna-12 and HC-1, suggesting they may represent EBV-transformed bystander B cells rather than malignant clones^23,24^. Additionally, the origins of MEC-1 and PGA-1 cell lines are uncertain. This incidental finding underscores the importance of rigorous cell line authentication.

Thus, considering the observed clonal evolution, and the presence of EBV in lcl_burn0214, we conclude that the examined cell line is more likely classified as lymphoblastoid rather than malignant lymphoma. However, the question of whether the EBV + lcl_burn0214 cell line originates from infiltrating B cells—which, in culture, lack the immune control mechanisms that would normally regulate EBV-driven proliferation—or alternatively from a lymphoproliferative process already present in the original rectal tumor biopsy, remains open. Supporting the hypothesis of immune control mechanism loss is the fact that the patient remained in remission without signs of lymphoproliferative disorder for at least four years after surgery.

On the one hand, EBV-infected B cells are recognized by T cells^25,26^; on the other hand, EBV is known to manipulate B cell metabolism. EBV-transformed immortalized B cells can exhibit immunomodulatory features that help them evade T cell–mediated immune responses and proliferate unchecked in immunocompromised hosts^1,18^. Nevertheless, the ability of EBV-transformed B cells to induce lymphomagenesis likely varies due to differences in their antigenicity and immunogenicity, which are particularly associated with variations in the EBV genome^27^. This has been demonstrated in animal studies that evaluated the tumor-forming ability of various B lymphocytic and lymphoblastoid cell lines, in different strains of immunocompromised mice^7–10,14^. In our study, the lcl_burn0214 cell line at passage 14 showed only transient, limited tumor growth in nude mice. It should be noted though that we used a small cohort of animals (*n* = 5), so low but true tumorigenic potential may have been missed. Although nude mice lack mature T cells, they retain T cell precursors. Other reports have demonstrated that EBV-infected B cells form tumors more readily in more severely immunocompromised mice (e.g., SCID, NOG)^7–10,14^. Also one study showed that lymphoblastoid B cells with trisomy 12 could initiate tumor growth in nude mice^20^. Thus, the tumorigenic potential of lcl_burn0214 though cannot be excluded at later passages or in more severely immunocompromised animals at earlier passages, remains low and is unlikely to pose an issue for outgrowth during autologous TILs generation in our case.

As previously mentioned, EBV-transformed B cells can evade T cell–mediated immune responses. Data from other studies show that approximately 0.01–0.1% of all PBMCs in seropositive healthy individuals are EBV-specific T cells. At the same time, patients with EBV-positive diffuse large B-cell lymphoma or systemic lupus erythematosus exhibit a significantly reduced number of such EBV-specific clones compared to healthy individuals^28,29^. In our study, we observed a frequency of EBV-specific TIL clones and an IFN-γ response comparable to that of healthy donors, both in response to EBV peptides and to the lcl_burn0214 cells themselves. Additionally, TILs_burn0214 were both B cell- and EBV-negative. This suggests that autologous TILs are capable of exerting immune control, preventing the proliferation of EBV + B cells present in the patient ex vivo and in vivo. We did not examine the potential for lcl_burn0214 to skew T cell receptor (TCR) repertoires toward EBV antigens in vivo. Nevertheless, previous studies have demonstrated a shift in TCR repertoires toward EBV-specific clones, accompanied by repertoire narrowing, notably during the generation of T cell products targeting EBV upon cocultivation with autologous lymphoblastoid cell lines^10,25^.

In conclusion, we report spontaneous arising of an immortalized wild-type EBV-infected B cell line, lcl_burn0214 derived from a rectal tumor specimen. Patients’ PBMCs and tumor specimens were found negative for EBV DNA, highlighting the difficulty in predicting the development of EBV-mediated lymphoproliferation both ex vivo and in vivo. In our case, the identification of EBV-specific T cell clones among autologous TIL_burn0214, along with the low tumorigenic potential of the lcl_burn0214 cell line, supports the notion that EBV-transformed B cells can be effectively controlled by the immune system in the context of autologous TIL transplantation. Consequently, we infer that in this patient, the likelihood of tumor-infiltrating EBV-transformed B cells evading immune surveillance—such as during periods of lymphodepletion therapy before the autologous TIL product infusion—is low, and unlikely to lead to EBV-driven proliferation or lymphoproliferative disorders in vivo. Things may differ in other patients whose B cells are infected with different EBV strains or whose TILs lack EBV-specific activity.

Our findings highlight the importance of investigating the relationships between EBV-transformed B-TILs and autologous TILs, as well as screening TIL products for B cells, EBV and the presence of EBV-specific T cell clones during manufacturing, to optimize therapeutic outcomes.

## Materials and methods

### Patient information

The patient was a 46-year-old Caucasian male diagnosed with microsatellite-stable, moderately differentiated (T4N0M0 G2) rectal adenocarcinoma. He presented with grade 3 arterial hypertension and grade 3 ulcerative disease of the duodenum as comorbid conditions. No treatment was administered prior to the surgical intervention. The patient survived for at least four years post-surgery and did not develop any subsequent lymphoproliferative disorders. The tumor sample (designated tum_burn0214) and the blood sample were obtained during surgery. For tumor and blood samples from a patient this study was approved by the local institutional review board of the Burnasyan Federal Medical Biophysical Center FMBA of Russia under protocol RU-FMBC-23-07-2020. The study was conducted in accordance with the Declaration of Helsinki. Informed consent was obtained from the patient prior to his participation.

### Tumor tissue and blood processing

The tumor sample tum_burn0214 was placed in DMEM supplemented with 1× antibiotic-antimycotic solution (Thermo Fisher Scientific, Waltham, MA USA) and maintained at 2–8 °C until processing, which occurred within 2 h of surgery. The sample was then cut into 1.5 × 1.5 mm fragments. Four fragments were used fresh to establish primary tumor cultures. A portion of the specimen was preserved in IntactRNA solution (Evrogen, Moscow, Russia) for subsequent RNA extraction, while the remaining fragments were cryopreserved in DMEM/F12 supplemented with 45% fetal bovine serum (FBS) and 10% dimethyl sulfoxide (DMSO) for tumor-infiltrating lymphocyte (TIL) expansion studies. Peripheral blood mononuclear cells (PBMCs) were isolated from whole blood using Ficoll-Hypaque density gradient centrifugation and cryopreserved in RPMI1640 containing 45% FBS and 10% DMSO. Cell viability and count were assessed immediately after isolation by trypan blue staining using a hemocytometer. PBMC viability was more than 95% immediately after isolation. After thawing, PBMC viability was more than 85%.

### Primary cell line establishment

Four fresh tumor fragments, tum_burn0214 (1.5 × 1.5 mm each), were enzymatically digested with 50 units/mL collagenase IV for 1 h. After washing with phosphate-buffered saline (PBS), the resulting cell suspension was placed in a 6-well culture plate containing DMEM/F12 medium supplemented with 10% FBS, 2 mM Glutamax, and 1× antibiotic/antimycotic, and maintained in a humidified atmosphere with 5% CO₂. The cell viability, assessed immediately after the digestion step by trypan blue staining and counted using a hemocytometer, was greater than 70%, although a significant amount of debris was present. The culture medium was replaced weekly. When the cell density reached 1–1.5 million cells per milliliter (cells/mL), the cells were split to a concentration of 0.2–0.3 million cells/mL and transferred to new culture flasks. Cryopreservation of the established cell line, lcl_burn0214, was performed at passages 6, 15, 35, 46, and 62 using DMEM/F12 supplemented with 45% FBS and 10% DMSO. Thawing tests were successfully conducted for all specified passages.

### Tumor-Infiltrating lymphocyte (TIL) TIL_burn0214 expansion

Thawed tumor fragments, tum_burn0214 (5–10 fragments per well of a 6-well plate), were placed in 2 mL of serum-free lymphocyte culture medium HIPP-T009 (Bioengine, Shanghai, China) supplemented with 5,000 units/mL IL-2 (Ronkoleukin, Biotech, Moscow, Russia) and 1× antibiotic/antimycotic (TIL culture medium). The ImmunoCult™ Human CD3/CD28 T Cell Activator (STEMCELL Technologies, Vancouver, BC, Canada) was added at a volume of 25 µL per 1 mL of total culture volume, as recommended by the manufacturer, to activate T cells on day 0. Reactivation with the T Cell Activator was performed twice during a 14-day culture period, with activations occurring on day 0 and day 10 in accordance with the manufacturer’s protocol. The TIL medium was refreshed two days post-activation. Upon reaching a density of 1–1.5 million cells/mL, TIL_burn0214 cells were subcultured into new flasks. After 14 days of culture, expanded TILs were subsequently characterized for phenotype and anti-EBV activity using the ELISPOT assay.

### In vivo tumorigenicity

The tumorigenic potential of lcl_burn0214 cells was evaluated in vivo by subcutaneous injection into five immunodeficient male NU–A/A Tyrc/Tyrc Foxn1nu/Foxn1nu mice (BALB/c nude strain, 6–8 weeks old; obtained from the Laboratory Animals Breeding Facility BIBCh, Pushchino, Russia). Animals were maintained under specific pathogen-free conditions in the Vivarium of the Department of Experimental Biology at the Shemyakin-Ovchinnikov Institute of Bioorganic Chemistry, Russian Academy of Sciences (IBCh RAS). Animal procedures were approved by the local institutional review board of the Vivarium of the Department of Experimental Biology at the IBCh RAS under protocol #378/2023 and carried out in accordance with the institutional guidelines. Lcl_burn0214 cells at passage 14 were harvested and resuspended in phosphate-buffered saline at a concentration of 1 × 10^7^ cells/mL. Each mouse received a subcutaneous injection of 0.1 mL cell suspension (total 1 × 10^6^ cells) into the right flank. Tumor growth was monitored weekly by measuring three orthogonal diameters (length, width, height) of the tumor using an electronic caliper. The tumor volume was calculated using the following formula: V = (π/6) * Length * Width * Height. Mice were euthanized 75 days post-injection. The only tumor that reached 200 mm³ was excised, fixed in 4% neutral-buffered formaldehyde. Samples fixed in 4% buffered formaldehyde were subjected to histological processing using isopropyl alcohol and xylene. The samples were embedded in blocks formed from Histomix Extra embedding medium (BioVitrum, Moscow, Russia). Tissue Sect. 3.5 μm thick were prepared. Hematoxylin-eosin staining was performed as previously described^30^. Additional staining methods, including immunohistochemistry, were not conducted.

### STR analysis

TIL_burn0214, PBMC_burn0214, the lcl_burn0214 cell line at passages 15, 53, from the patient were subjected to STR analysis to authenticate the cell line origin and exclude potential cross-contamination. 27 loci were amplified using PowerPlex^®^ Fusion 6 C System STR and detected by the Applied Biosystems 3500 Series Genetic Analyzer. Data were analyzed with GeneMapper ID‑X Software v1.6.

The STR profile of the lcl_burn0214 cell line was compared with Release 52 of April 2025 of the Cellosaurus database to identify any related cell lines using the CLASTR 1.4.4 online tool^31^.

### EBV testing

EBV testing of PBMC_burn0214, lcl_burn0214 at passage 53, TIL_burn0214, and tum_burn0214 samples was performed in a clinical laboratory using a validated qualitative assay. The EBARPOL kit (Litekh, Moscow, Russia) based on real-time polymerase chain reaction method was used. EBV status of the established lcl_burn0214 cell line at passage 14 and tum_burn0214 samples was additionally analyzed by gene expression in RNA-seq data.

### Karyotyping

For karyotyping analysis, nuclei suspensions of lcl_burn 0214 cells at passages 6 and 62 were applied to glass slides and processed according to the previously described method^32^. Eleven GTG-banded metaphase spreads were analyzed in terms of karyotype formula, GTG-stained karyotypes were recorded as per the International System for Human Cytogenomic Nomenclature^33^.

### Flow cytometry

Phenotypic analysis was performed on lcl_burn0214 and TIL_burn0214 cell cultures using the following monoclonal antibodies purchased from Sony Biotechnology, San Jose, Ca, USA: CD3_FITC (2186530), CD19_APC (2111055), and CD45_FITC (2120025), CD4_APC-Cy7 (174821), CD8_PE (2105255), CD20_PE (2111525). DAPI was used for dead cell discrimination. Cells (1 × 10^5^) were washed in PBS and resuspended in a staining buffer containing the appropriate antibodies. Surface marker staining was performed for 20 min at 4 °C. After incubation, cells were washed twice, collected in 100 µl of PBS, and immediately analyzed using the ACEA NovoCyte^®^ flow cytometer VBR system (Agilent Technologies, Santa Clara, CA, USA). NovoCyte Express software was used for plot analysis. For lcl_burn0214 phenotyping, a total of 20,000 events was gathered, with a threshold larger than 100,000 FCS-H, resulting in more than 7,000 events in the cell gate. For TIL_burn0214 phenotyping, a total of 60,000 events was gathered, with more than 40,000 events in the cell gate. The gating strategy included gating on all events to remove debris (FSC-A vs. SSC-A), excluding doublets (FCS-H vs. FSC-A), and excluding dead cells by gating on DAPI-negative events.

### Elispot assay

To evaluate the functional response of tumor-infiltrating lymphocytes (TILs) to the EBV-positive autologous lcl_burn0214 cell line, co-culture experiments were performed. The TIL_burn0214 and lcl_burn0214 cell line were co-cultured at effector-to-target (E: T) ratios of 1:1 and 1:10 in 96-well flat-bottom plates pre-coated with anti-IFN-γ antibodies. Each well contained either 5 000 or 50 000 TIL_burn0214 cells and 50 000 lcl_burn0214 cells. TIL_burn0214 cells were thawed and used immediately in the assay. Two vials of TIL_burn0214 were utilized as biological replicates. For each vial, co-culture conditions were run in duplicate wells, resulting in two technical replicates per biological replicate.

After 18 h of incubation, IFNγ production was quantified using a commercially available ELISPOT kit (ImmunoSpot^®^ ELISPOT kit, Cellular Technology Limited, C.T.L., Cleveland, OH, USA), following the manufacturer’s protocol. Negative controls included wells containing either 50 000 lcl_burn0214 or 50 000 TIL_burn0214 cultured alone. Developed ELISPOT plates were analyzed using the Immunospot analyzer software (C.T.L.), and the mean number of IFN-γ spot-forming units (SFUs) was calculated for each condition: TIL_burn0214 co-cultured with lcl_burn0214, TIL_burn0214 alone, and lcl_burn0214 alone.

To evaluate the TIL response to EBV peptides, 80 000–200 000 TIL_burn0214 cells were incubated for 48 h with or without the EBV peptide pool (EBV HLA Class I Control Peptide Pool, STEMCELL Technologies, Vancouver, BC, Canada) at a final concentration of 2 µg/mL for each peptide in the pool. IFNγ production was quantified using a commercially available ELISPOT kit as described above. The assay was performed using two vials of TIL_burn0214 as biological replicates, each in duplicate wells of a 96-well ELISPOT plate provided with the kit.

Two-sided Kruskal-Wallis rank test was used to assess differences among the testing groups in the ELISPOT assay.

### RNA extraction and construction of transcriptome libraries

RNA extraction from the following samples: tumor tissue tum_burn0214 and the cell line lcl_burn0214 at passage 14 was performed using the All Prep RNA/DNA Mini Kit (Qiagen, Hilden, Germany). Tissue fragments preserved in Intact RNA solution were minced on a sterile Perti dish until they were as small as possible. Tissue fragments were then transferred to a 2 ml tube containing three stainless steel spheres of 4.8 mm diameter, to which 700 µL of RLT lysis solution containing 1% beta-mercaptoethanol was added. Tissue fragments were homogenized using the Tissue Lyser instrument (Qiagen, Hilden, Germany) under the following conditions: 1 min, 25/s. The next steps of RNA extraction were performed according to the manufacturer’s instructions for animal tissues. RNA from lcl_burn0214 was also isolated using the All Prep RNA/DNA Mini Kit (Qiagen, Hilden, Germany) according to the manufacturer’s instructions for animal cells. Approximately 4*10^6^ fresh pelleted cells were used as input. Homogenization was performed using a 20G needle with a syringe. Total RNA integrity number (RIN) was determined using RNA 6000 Nano Kit (Agilent, Santa Clara, CA, USA) with Bioanalyzer 2100 instrument (Agilent, USA). TURBO DNA-free Kit (Thermo Fisher Scientific, Waltham, MA, USA) was used to remove DNA traces. For the construction of the transcriptome library, 500 ng of total RNA was collected. First, RNA was enriched for poly(A) fraction using NEBNext Poly(A) mRNA Magnetic Isolation Module (New England Biolabs, Ipswich, MA, USA). Next, non-directional transcriptome libraries were prepared using the NEBNext Ultra II RNA Library Prep Kit for Illumina (New England Biolabs, USA) with the NEBNext Multiplex Oligos for Illumina (Dual Index Primers Set 1) (New England Biolabs, USA) according to the manufacturer’s instructions. Paired-end libraries were sequenced on the Illumina NovaSeq 6000 instrument at 2 × 100 cycles (Illumina, San Diego, CA, USA).

### Bioinformatics pipeline

#### Data Preparation for analysis

Lymphoma cell line transcriptomic data (Quentmeier H. dataset)^34^, were downloaded from ENA (PRJEB30312)^35^, B95-8 EBV strain-infected lymphoblastoid B cell lines (Geuvadis consortium dataset)^36^ were downloaded from IGSR^37^. Quality control by FastQC (0.12.1)^38^ and MultiQC (1.15)^39^ was performed before and after adapter trimming by cutadapt (version 4.4)^40^ and quality filtering by trimmomatic (version 0.39)^41^ for the whole data set. Pseudoalignment was performed by salmon (1.10.2)^42^ for Gencode.v43 transcriptome with full genome decoy index. Summarizing for gene was performed by tximport (1.26.1)^43^ using the length scaled TPM metrics and ignoring transcripts version. Expression and dispersion for genes and transcripts has been estimated by edgeR (version 3.40.2)^44^. For further analysis, CPM data from edgeR was used, filtered by a maximum mean of greater than 4 CPM in any group and limited to protein-coding genes. We used normalized expression values in CPM to provide unified and comparable metrics for clustering and correlation across different sample types. Dispersion was simultaneously evaluated during the CPM calculation using edgeR. PCA analysis was realized by PCAtools (v2.10.0)^45^ on log2 transformed CPM removing genes with the lowest 10% variance. Pearson correlation matrices were used to compare different samples. A mixed-effects model with clusters/subgroups as moderators was applied to explain the variability in correlations using the metafor package https://www.jstatsoft.org/article/view/v036i03. Confidence intervals were set at 95%. ANOVA tests were employed to evaluate models with different moderators. Plots were plotted with ggplot2 (v3.4.3)^46^.

Deconvolution of cell types were realized by MSP, xCell and EPIC methods using the immunedeconv R package (v2.1.3)^47^ with TPM data.

### HLA typing for RNA-seq data (tum_burn0214 and lcl_burn0214 samples)

HLA typing was performed with HLA-HD^48^ against IMGT/HLA database (version 3.50)^49^ using RNA-seq data from tum_burn0214 and lcl_burn0214 samples.

#### EBV reference genome aligning

RNA-seq reads were aligned to the human reference genome using STAR version 2.7.10b^50^ in 2-pass mode. The reference genome used was the GRCh38 (hg38) assembly provided by the GDC^51^, which includes decoy and 200 viral genomes including the EBV; the genome index for STAR was generated using GENCODE v43 gene annotations. The reference genome EBV sequence is the type 1 EBV surrogate genome sequence (NC_007605), constructed based on the B95-8 and Raji EBV genomes^52^.

### BCR profiling analysis

BCR repertoire were evaluated by MiXCR (version 4.6.0)^53^.

## Supplementary Information

Below is the link to the electronic supplementary material.

Supplementary Material 1

## Data Availability

RNA-seq data produced in this study have been deposited at the BioProject and are available under accession number PRJNA1266483.
