# Supplementary material for: Latent EBV reactivation drives aberrant B-cell proliferation during ex vivo tumor-infiltrating lymphocyte expansion from EBV-negative rectal cancer tumor tissue
Source: Sci Rep. 2025 Nov 24;15:45250. doi: 10.1038/s41598-025-29456-7 (PMC12749248; doi:10.1038/s41598-025-29456-7)
Supplement: Supplementary file 1 — Supplementary Material 1 [file 41598_2025_29456_MOESM1_ESM.docx]

Latent EBV reactivation drives aberrant B-cell proliferation during ex vivo tumor-infiltrating lymphocyte expansion from EBV-negative rectal cancer tumor tissue

Tatiana V. Petrova^1^*, Daria V. Kuznetzova^1^, Alexandra V. Kanygina^1^, Liubov O. Skorodumova^1^, Viktor A. Ivanov^1^, Tatiana A. Astrelina^2^, Svetlana E. Varlamova^2^, Elena A. Zerkalenkova^3^, Elena I. Sharova^1^*

*Correspondence: Tatiana V. Petrova, petrozik@gmail.com Elena I. Sharova [sharova78@gmail.com](mailto:sharova78@gmail.com)

^1^ Lopukhin FRCC PCM;

^2^ The Burnasyan Federal Medical Biophysical Center FMBA of Russia;

^3^ Dmitry Rogachev National Scientific and Practical Center of Pediatric Hematology, Oncology and Immunology, Moscow, Russia

**Table of contents**

**Supplementary Figures**

**Supplementary Tables**

**Supplementary Figures**

A


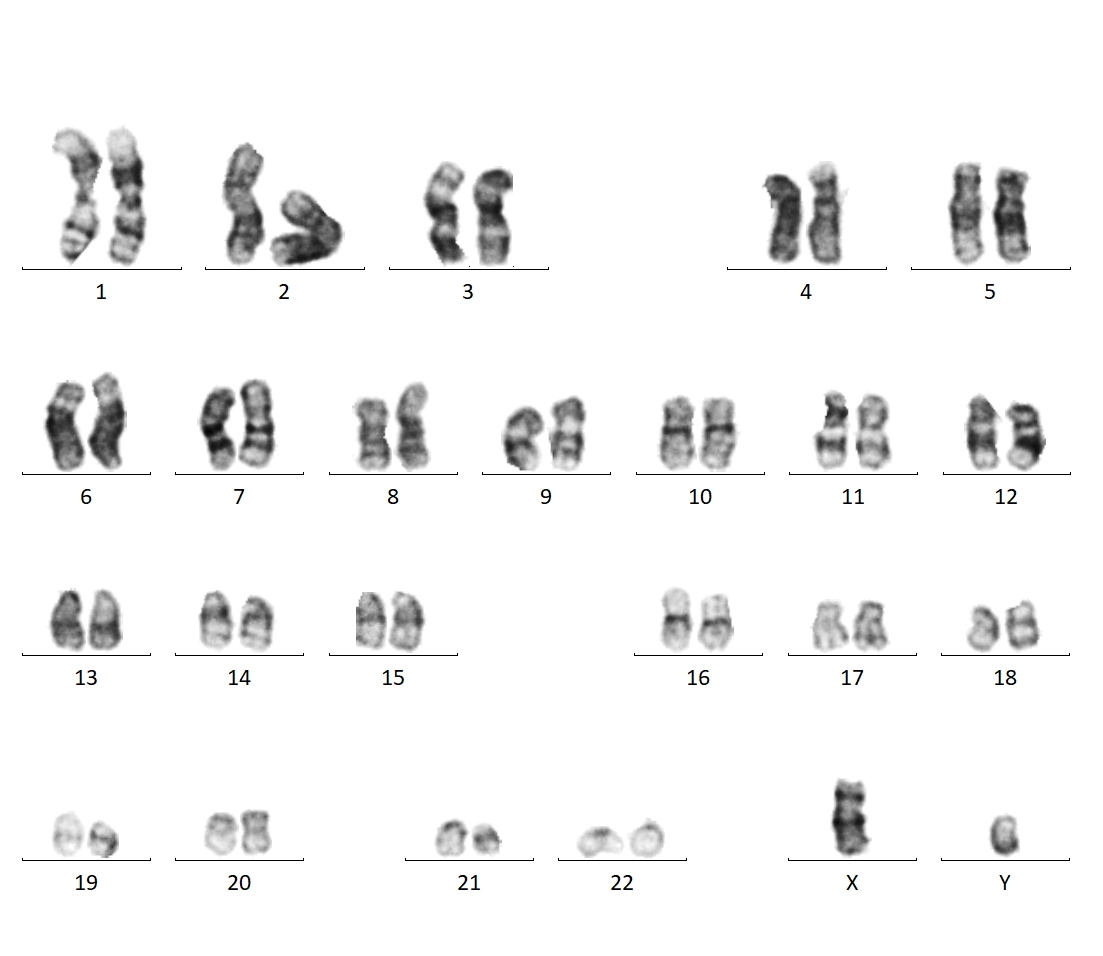


B
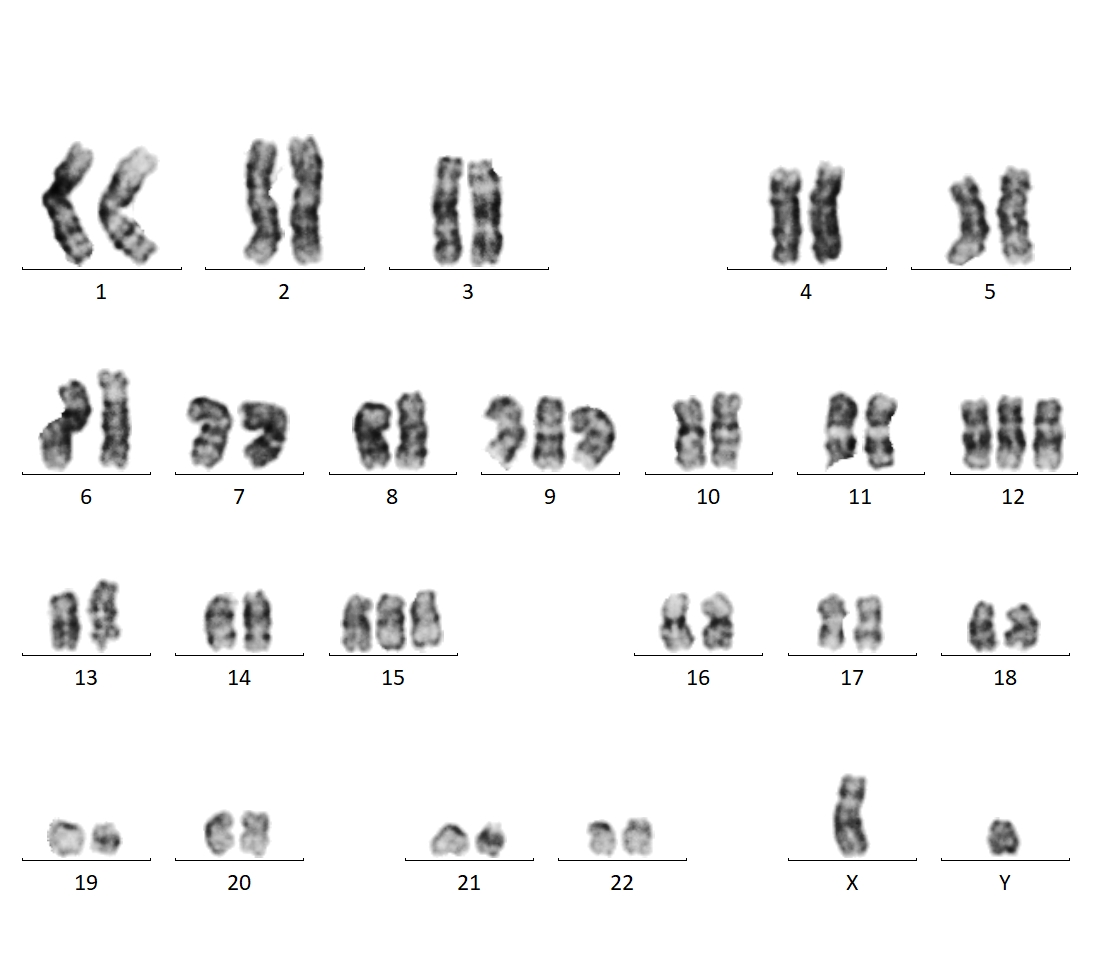


## Supplementary Figure 1. Karyotype analysis of the lcl_burn0214 cell line at passages p6 and p62

A Karyotype analysis of the lcl_burn0214 cell line at passage p6. 46 XY karyotype

B Karyotype analysis of the lcl_burn0214 cell line at passage p62. 49XY karyotype with trisomy of chromosomes 9, 12, 15.

**
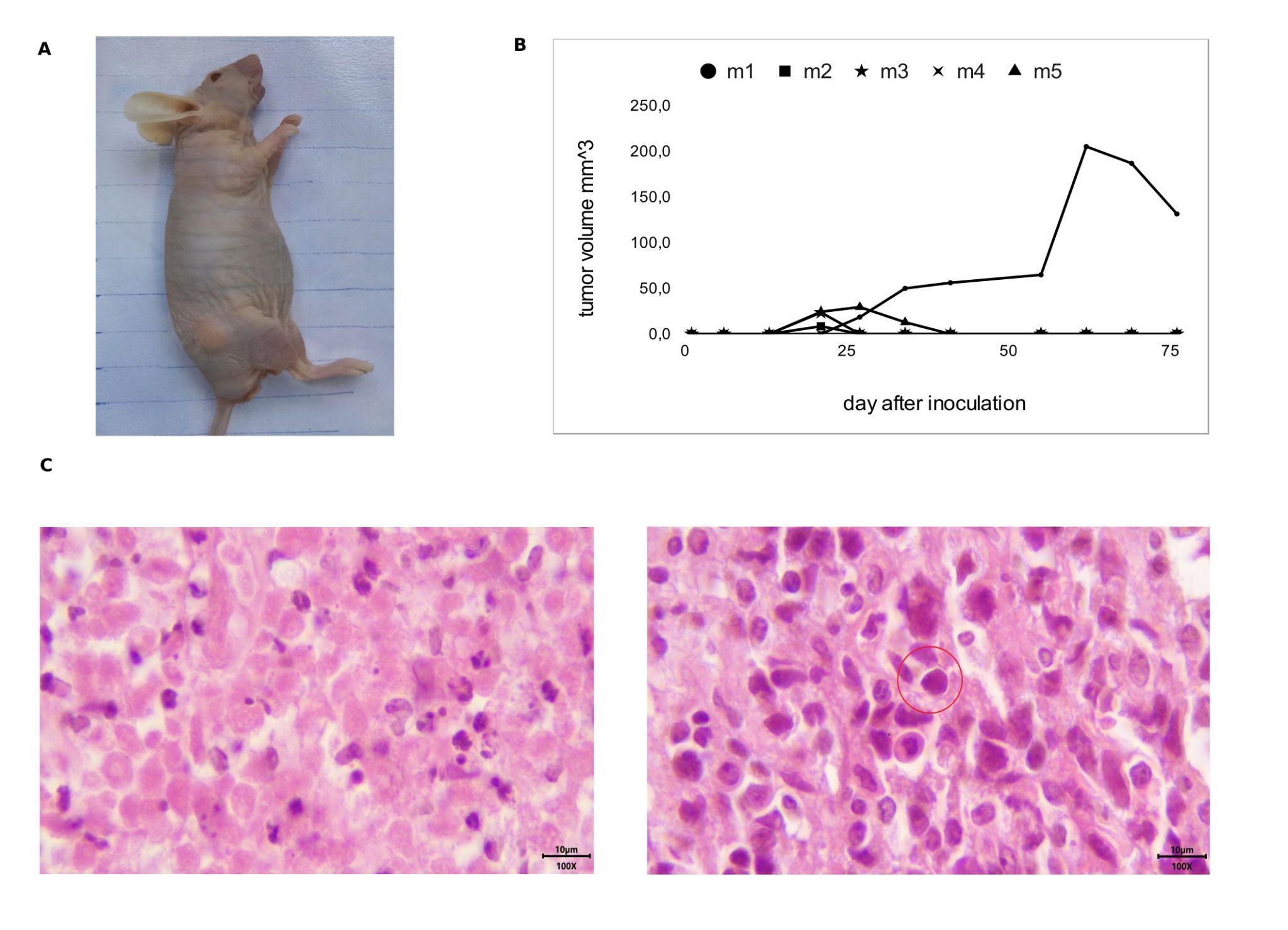
**

**Supplementary Figure 2. Tumorigenicity of the lcl_burn0214 cell line in NU–A/A Tyrc/Tyrc Foxn1nu/Foxn1nu mice.**

A Representative image of the tumor-bearing mouse.

B Tumor growth curve. One million cells of lcl_burn0214 cell line were subcutaneously inoculated into 5 NU–A/A Tyrc/Tyrc Foxn1nu/Foxn1nu male mice (m1-m5). Graft growth was observed in four out of five mice and was monitored over the subsequent 75 days. Three (m2, m3, m4) out of four lcl_burn0214 grafts regressed over a period of 34 days, with the exception of the tumor in mouse m1.

C Hematoxylin and eosin staining of tumor graft section from m1 mice (tissue sections are 3.5 μm thick). The xenograft tumor exhibited large zones of necrosis with few alive blasts, the blast on the representative image is highlighted with a red circle (scale bar = 10 μm, 100x magnigication).

## Supplementary Tables

## Supplementary Table 1. Number of BCR clonotypes and proportion of the most frequent BCR clonotype in tum_burn0214 and lcl_burn0214 samples

| BCR chain name | tum_burn0214  Number of clonotypes/proportion of the most frequent BCR clonotype | lcl_burn0214  Number of clonotypes/proportion of the most frequent BCR clonotype |
| --- | --- | --- |
| IGH | 176/0.12 | 16/.92 |
| IGK | 305/0.28 | 14/0.99 |
| IGL | 180/0.1 | 8/0.98 |

##

## Supplementary Table 2A. Cell deconvolution analysis of tum_burn0214 and lcl_burn0214 samples using bulk RNA-seq data and EPIC method

| cell entity | lcl_burn0214 | tum_burn0214 |
| --- | --- | --- |
| **B cell** | **0,43** | **0,00** |
| Cancer associated fibroblast | 0,00 | 0,01 |
| T cell CD4+ | 0,00 | 0,01 |
| T cell CD8+ | 0,00 | 0,01 |
| Endothelial cell | 0,00 | 0,01 |
| Macrophage | 0,00 | 0,00 |
| NK cell | 0,00 | 0,00 |
| uncharacterized cell | 0,57 | 0,95 |

## **Supplementary Table 2B.** Cell deconvolution analysis of tum_burn0214 and lcl_burn0214 samples using bulk RNA-seq data and xCell method

| cell entity | lcl_burn0214 | tum_burn0214 |
| --- | --- | --- |
| Myeloid dendritic cell activated | 0,00 | 0,00 |
| **B cell** | **1,01** | **0,00** |
| T cell CD4+ memory | 0,01 | 0,00 |
| T cell CD4+ naive | 0,00 | 0,00 |
| T cell CD4+ (non-regulatory) | 0,00 | 0,00 |
| T cell CD4+ central memory | 0,00 | 0,00 |
| T cell CD4+ effector memory | 0,00 | 0,00 |
| T cell CD8+ naive | 0,00 | 0,00 |
| T cell CD8+ | 0,00 | 0,00 |
| T cell CD8+ central memory | 0,00 | 0,00 |

## **Supplementary Table 2C.** Cell deconvolution analysis of tum_burn0214 and lcl_burn0214 samples using bulk RNA-seq data and MCP method

| cell entity | lcl_burn0214 | tum_burn0214 |
| --- | --- | --- |
| T cell | 1,49 | 2,18 |
| T cell CD8+ | 0,00 | 4,12 |
| cytotoxicity score | 8,65 | 2,34 |
| NK cell | 0,01 | 0,14 |
| **B cell** | **1141,69** | **30,40** |
| Monocyte | 10,82 | 3,49 |
| Macrophage/Monocyte | 10,82 | 3,49 |
| Myeloid dendritic cell | 1,01 | 1,14 |
| Neutrophil | 6,96 | 3,92 |
| Endothelial cell | 2,67 | 3,76 |

## Supplementary Table 3. Short tandem repeat profiles of the PBMC_burn0214, TIL_burn0214 and lcl_burn0214 cell line at passages 15 and 53

| **Locus** | **PBMC_ burn0214** | **TIL_burn0214** | **lcl_burn0214_p53** | **lcl_burn0214_p15** |
| --- | --- | --- | --- | --- |
| Amelogenin | X;Y | X;Y | X;Y | X;Y |
| D3S1358 | 17 | 17 | 17 | 17 |
| D1S1656 | 15;16 | 15;16 | 15;16 | 15;16 |
| D2S441 | 11 | 11 | 11 | 11 |
| D10S1248 | 14;16 | 14;16 | 14;16 | 14;16 |
| D13S317 | 9;11 | 9;11 | 9;11 | 9;11 |
| Penta E | 10;12 | 10;12 | 10;12 | 10;12 |
| D16S539 | 10;12 | 10;12 | 10;12 | 10;12 |
| D18S51 | 14;16 | 14;16 | 14;16 | 14;16 |
| D2S1338 | 23;26 | 23;26 | 23;26 | 23;26 |
| CSF1PO | 11 | 11 | 11 | 11 |
| Penta D | 12 | 12 | 12 | 12 |
| TH01 | 6 | 6 | 6 | 6 |
| vWA | 16;17 | 16;17 | 16;17 | 16;17 |
| D21S11 | 32.2 | 32.2 | 32.2 | 32.2 |
| D7S820 | 10 | 10 | 10 | 10 |
| D5S818 | 12;13 | 12;13 | 12;13 | 12;13 |
| TPOX | 8;11 | 8;11 | 8;11 | 8;11 |
| D8S1179 | 11;12 | 11;12 | 11;12 | 11;12 |
| D12S391 | 19;21 | 19;21 | 19;21 | 19;21 |
| D19S433 | 12;15 | 12;15 | 12;15 | 12;15 |
| SE33 | 18;30.2 | 18;30.2 | 18;30.2 | 18;30.2 |
| D22S1045 | 15;16 | 15;16 | 15;16 | 15;16 |
| DYS391 | 10 | 10 | 10 | 10 |
| FGA | 20;21 | 20;21 | 20;21 | 20;21 |
| DYS576 | 19 | 19 | 19 | 19 |
| DYS570 | 18 | 18 | 18 | 18 |

##

## **Supplementary Table 4.** HLA genotype of the tum_burn0214 and lcl_burn0214 samples

HLA genotype: A03:01:01, A03:01:01, B07:02:01, B40:02:01, C02:02:02, and C07:02:01

| HLA gene | A | A | B | B | C | C |
| --- | --- | --- | --- | --- | --- | --- |
| HLA allele | A03:01:01 | A03:01:01 | B07:02:01 | B40:02:01 | C02:02:02 | C07:02:01 |

## Supplementary Table 5. EBV status of the tested samples using Real-time PCR analysis

| sample name | EBV status positive/negative |
| --- | --- |
| PBMC_burn0214 | negative |
| TIL_burn0214 | negative |
| lcl_burn0214 at passage 53 | positive |
| tum_burn0214 | negative |

## Supplementary Table 6. Characteristics of selected B cell lines from Quentmeier H. dataset

| Entity | Cell line name | Selected aberrations | Sex | Age | Ethnicity | EBV status | ERR # |
| --- | --- | --- | --- | --- | --- | --- | --- |
| B-NHL: PLL | MEC-1 | R3HCC1L-HTRA1 | M | 61 | european | positive | ERR3003563 |
| B-NHL: PLL | PGA-1 |  | M | n.d. | caucasian | positive | ERR3003584 |
| B-NHL: DLBCL | NU-DHL-1 | t(3;8)(p25;q24); t(14;18)(q32;q21) | M | 73 | european | negative | ERR3003577 |
| B-NHL: DLBCL | DOHH-2 | t(8;14;18)(q24;q32;q21) | M | 60 | european | negative | ERR3003528 |
| B-NHL: HCL | BONNA-12 |  | M | 46 | european | positive | ERR3003517 |
| B-NHL: HCL | HC-1 |  | M | 56 | european | positive | ERR3003535 |
| B-NHL: mantle cell lymphoma | REC-1 | t(11;14)(q13;q32) | M | 61 | european | negative | ERR3003587 |
| B-NHL: PEL | BC-3 |  | M | 85 | european | negative | ERR3003514 |
| B-NHL: PEL | CRO-AP2 |  | M | 49 | european | positive | ERR3003522 |
| Hodgkin Lymphoma | HDLM-2 |  | M | 64 | european | negative | ERR3003536 |

B-cell non-Hodgkin lymphoma (B-NHL); hairy cell leukemia (HCL); primary effusion lymphoma (PEL); prolymphocytic leukemia (PLL); diffuse large B cell lymphoma (DLBCL)

## Supplementary Table 7. Restricted maximum likelihood (REML) model features for measurement of the correlation of lcl_burn0214 with the groups of B95-8_LCLs, HL cell line, and Non-Hodgkin lymphoma B_NHL cell lines

| model type | df | AIC | BIC | AICc | logLik | LRT | pval | QE | tau.2 | R.2 |
| --- | --- | --- | --- | --- | --- | --- | --- | --- | --- | --- |
| full | 6 | -59.70102 | -55.06549 | -50.36769 | 35.85051 | NA | NA | 114.0921 | 0.0005201562 | NA |
| reduced | 5 | -41.09325 | -36.92718 | -35.63871 | 25.54663 | 20.60777 | 5.636681e-06 | 420.3016 | 0.0027441086 | 81.04462 |

degrees of freedom (df); Akaike Information Criterion (AIC); Bayesian Information Criterion (BIC); the Akaike Information Criterion, corrected for small sample sizes (AICc); likelihood log (loglik); Likelihood Ratio Test (LRT); Quadratic Estimator (QE); proportion of variance (R.2). The best model chosen for measuring the correlation is the full model. The full model includes the following groups or samples: the B95-8_LCL group; the LBC_susp group, consisting of Bonna-12, HC-1,MEC-1, PGA-1 cell lines; the B_NHL group, consisting of the REC-1, NU-DHL-1, DOHH-2, BC-3, and CRO-AP2 cell lines; the HL cell line HDLM-2; and the tum_burn0214 sample.

The reduced model includes the following groups or samples: the B95-8_LCL group; the B_NHL group, consisting of the REC-1, NU-DHL-1, DOHH-2, BC-3, and CRO-AP2 along with Bonna-12, HC-1, MEC-1 and PGA-1 cell lines; the HL cell line HDLM-2; and the tum_burn0214 sample.

## Supplementary Table 8. List of EBV peptides potentially specific to TIL_burn0214

| peptide sequence | EBV gene | HLA allele |
| --- | --- | --- |
| RVRAYTYSK | EBV BRLF1 148 - 156 | HLA-A*03:01 |
| RLRAEAQVK | EBV EBNA-3A 603 - 611 | HLA-A*03:01 |
| RPPIFIRRL | EBV EBNA-3A 247 - 255 | HLA-B*07:02 |
| QPRAPIRPI | EBV EBNA-3C 881 - 889 | HLA-B*07:02 |

## 
